# The effects of esketamine on blood pressure and hypotension incidence during induction of bariatric surgery: A randomized controlled trial

**DOI:** 10.1097/MD.0000000000036754

**Published:** 2023-12-22

**Authors:** Ting Yang, Muhammad Saqib Mudabbar, Mingxing Xu, Qingmei Xiang, Bin Liu, Qiang Fu

**Affiliations:** a Department of Anesthesiology, The Third People's Hospital of Chengdu Affiliated to Southwest Jiaotong University, Chengdu, Sichuan, China; b Department of Cardiovascular Medicine, West China Hospital, Sichuan University, Chengdu, Sichuan Province, China; c Department of Anesthesiology, West China Hospital, Sichuan University, Wuhou District, Chengdu, Sichuan Province, China.

**Keywords:** bariatric surgery, esketamine, hypotension, induction phase, postoperative pain

## Abstract

**Background:**

The prevalence of obesity is high. Bariatric surgery is an effective treatment for severe obesity; however, the induction phase of anesthesia in these patients poses a risk of hypotension. Esketamine, known for its sympathetic nervous system stimulation, may stabilize blood pressure during induction. This study aimed to investigate the effects of esketamine on blood pressure in bariatric surgery patients.

**Methods:**

This randomized controlled trial included 145 patients undergoing bariatric surgery. Patients were randomly assigned to receive esketamine or a control intervention during induction. Blood pressure and other vital signs were measured and compared between the 2 groups using statistical analyses.

**Results:**

Administration of esketamine increased blood pressure before intubation (T2). The incidence of hypotension was lower in the esketamine group at multiple time points during induction. Postoperatively, the esketamine group exhibited lower pain scores at 24 hours and a reduced need for rescue analgesics.

**Conclusion:**

A single dose of 0.2 mg/kg esketamine during the induction phase of bariatric surgery can improve blood pressure stability and decrease the incidence of hypotension. Furthermore, it is associated with reduced postoperative pain. Future studies could explore the effects of higher esketamine doses and validate these findings in a larger and more diverse patient population.

**Preexisting insights**

□ Obese patients have incidences of low blood pressure due to postural change during bariatric surgery after induction.
□ Anesthetics also cause a decrease in blood pressure.
□ Esketamine in low doses helps stabilize blood pressure.

**Key discoveries**

□ A single dose of 0.2 mg/kg esketamine during the induction phase of bariatric surgery can:
□ Improve blood pressure stability and decrease the incidence of hypotension.
□ Reduce postoperative pain.

**How might your results change the direction of research or the focus of clinical practice?**

□ During obesity surgery, anesthesiologists could start giving a single dose of esketamine before bariatric surgery to patients to improve their hemodynamic stability and decrease the chances of incidences of hypotension.

## 1. Introduction

Obesity is a prevalent global health concern,^[1]^ and bariatric surgery has emerged as a safe and effective approach to address severe obesity with substantial and long-lasting weight loss.^[2]^ Sleeve gastrectomy and Roux-en-Y gastric bypass are among the widely utilized bariatric procedures, known for their efficacy and relatively low complication rates.^[3]^ However, with any surgical procedure, there are risks involved, and one of the critical challenges is the management of hemodynamic stability, particularly during the induction phase of anesthesia.^[4]^ Hypotension, which is generally recognized as blood pressure below 90/60 mm Hg,^[5]^ is a recognized concern during this phase of bariatric surgery.^[6]^ Hypotension during the induction phase of anesthesia is related to organ injury,^[7,8]^ intraoperative hypotension,^[9]^ and is associated with other poor postoperative prognosis.^[10,11]^ Patients undergoing bariatric surgery face an elevated risk of hypotension due to their preexisting cardiovascular vulnerabilities, often manifested as hypertension.^[12]^ This association is frequently observed in patients with metabolic syndrome, and they often opt for bariatric surgery as a means to address not only obesity but also hypertension and other metabolic disorders.^[13]^ As a result, upon induction, with the use of general anesthetics, the mechanism of action of which is through the nervous system, the blood pressure shows a significant decrease.^[6]^ To mitigate this risk, a pharmacological remedy such as esketamine, which is an antagonist of the N-methyl-D-aspartate receptor, is sometimes used in low doses before induction to avoid propofol injection pain and is known to increase blood pressure as a side effect.^[14,15]^ Previous studies observed that this effect of esketamine can be used to improve hemodynamic stability in patients undergoing procedures under general anesthesia.^[15–22]^ In light of the evidence from previous studies, we hypothesize that esketamine may improve blood pressure during the induction phase of the procedure since it stimulates the sympathetic nervous system, making it an ideal drug for the induction of anesthesia in unstable patients.^[23,24]^

### 1.1. Objective

To explore the effect of esketamine on improving hemodynamic stability during the induction phase of anesthesia.

## 2. Method

This study was a single-center double-blinded randomized controlled clinical trial. Prior to participation in this study, all subjects were asked for written informed consent for participating in the trial. The trial was registered prior to patient enrollment with the Chinese Clinical Trial Registry (ChiCTR2100054038, Principal investigator: Ting Yang, https://www.chictr.org.cn/com/25/showproj.aspx?proj=142806, Date of registration: December 7, 2021). This is a secondary study that shares the same trial registration number (ChiCTR2100054038) as a previously published study by Yang et al (2023).^[25]^

### 2.1. Patient enrollment

Patients enrolled in this study were recruited between March 2022 and March 2023. The inclusion criteria were patients between ≥18 years old who were undergoing bariatric surgery under general anesthesia at The Third People’s Hospital of Chengdu. Patients with American Society of Anesthesiologists physical status 1 to 3, BMI (body mass index) ≥ 27 kg/m^2^. The exclusion criteria were (1) patients who had bariatric surgery before; (2) patients with contraindications that did not allow for esketamine administration; (3) patients with glaucoma; (4) patients with a history of drug abuse including but not limited to opioids, amphetamines, ketamine, etc.

### 2.2. Sample size calculation

The minimal sample size was calculated according to our unpublished preliminary study with 30 patients, which showed that the mean of mean arterial blood pressure (MAP) taken right before intubation was 79.2 (±10.1) in the esketamine group and 72.9 in the control group. We set α = 0.05, 1-β = 0.95, and used the “Sample Size Calculator” to calculate the sample size online^[26]^; the total sample size was 134. Considering the loss to follow-up rate of 10%, it was estimated that 147 cases need to be included.

### 2.3. Randomization and blinding

Patients who met the inclusion criteria were assigned randomly into 2 groups with a ratio of 1:1. Randomization was done using random numbers created in SPSS 26.0 (IBM, Chicago, IL). Seventy-three patients were assigned to the control group, and 74 were assigned to the esketamine group. A biostatistician who was blinded to patients prepared sealed opaque envelopes for each patient. Before the surgery, independent researchers who did not participate in the analysis opened the envelopes and assigned each patient according to the envelope and prepared the placebo (normal saline—identical in appearance) or esketamine in syringes according to the assigned group of the patient. Anesthesiologists then unknowingly, administered the placebo or esketamine to the patient, blinded to the contents of the syringe.

### 2.4. Procedures

The patients were brought into the operation room, where the anesthesiologist connected the patient to a patient monitor (Biolight Anyview P22, Guangdong Biolight Meditech Co. Ltd., Zhuhai) to monitor the blood oxygen saturation, noninvasive blood pressure, bispectral index, and 3 lead continuous electrocardiography. Once the patients were connected to a patient monitor, an infusion line was placed to administer crystalloid infusion 5 mL/kg and penehyclidine hydrochloride 0.01 μg/kg bolus, followed by dexamethasone 10 mg bolus, and dexmedetomidine 0.5 µg/kg for 10 minutes, after which a blood pressure reading was recorded, this timestamp was labeled T1, followed by a continuous infusion of dexmedetomidine 0.2 μg/kg/h, until the gastric tissue resection, for sedation.

After the initial preparation procedures, the process of induction began, and the anesthesiologist unknowingly administered a placebo to the control group and the esketamine to the esketamine group; the following procedures were identical among the 2 groups.

A bolus dose of anesthetic drugs i.e., midazolam 0.03 mg/kg, sufentanil 0.3 μg/kg, etomidate 0.2 mg/kg, propofol 1 mg/kg, and rocuronium bromide 0.9 mg/kg were administered. Following this, when induction drugs began effect, a second blood pressure measurement was taken right before intubation; this timestamp was labeled as T2. Then, 1 minute and 5 minutes after intubation, 2 readings were taken, and the timestamps were labeled as T3 and T4, respectively. Then, a target-controlled infusion of propofol, remifentanil, and sevoflurane, which were adjusted with objective monitoring BIS, maintaining it between 40 to 60, followed by nerve block, that is, 10 mL of 0.5% ropivacaine for the bilateral rectus abdominalis sheath block and 0.25% ropivacaine 20 mL for the unilateral right transversalis fascia block administered locally. The patients then underwent gastroscopy to evaluate their upper gastrointestinal tract and identify any potential issues or abnormalities before proceeding with bariatric surgery.

Three blood pressure readings were taken at the time of incision, 1 minute after incision and 5 minutes after incision; these timestamps were labeled T5, T6, and T7, respectively. 5 mg of tropisetron hydrochloride and 0.1 mg/kg of oxycodone was administered following gastric tissue resection. After the surgical procedure was concluded, anesthetic drugs were discontinued at the same time as suturing, which was followed by extubation, and patients were transferred to the postanesthesia care unit and then transferred to the general ward. All drugs administered based on body weight were titrated according to the adjusted body weight of the patients.

### 2.5. Outcome measures

Trial data were collected by anesthesiologists who were blinded to group assignment, and they were verified against patients’ electronic medical records. The baseline data that was collected included Age, Sex, Height, Weight, BMI, American Society of Anesthesiologists Classification,^[27]^ and Surgery Type: I referred to sleeve gastrectomy, and II referred to Roux-en-Y bypass. Intraoperative data included Surgery Duration, Extubation Time, Crystalloid Infusion, Colloid Infusion, Vasopressor Drugs, Drug Use Duration, Anti-Antagonist Drugs, and Incidence of Low Blood Pressure from T1 to T7. Postoperative data included: numerical rating scale (NRS) Score of 24th hour, Extra Pain Killers, Infusion Volume, Nausea and Vomiting Life Scale Score, and Abdominal Discomfort.

The primary outcome was blood pressure right before intubation, systolic blood pressure (SBP), diastolic blood pressure (DBP), and MAP at T2. Secondary outcomes included (1) SBP, DBP, and MAP measurements taken at T1, T3, T4, T5, T6, and T7. (2) Heart rate (HR) readings taken from T1 to T7. (3) Surgery Duration. (4) Extubation Time. (5) Crystalloid Infusion Volume. (6) Colloid Infusion Volume. (7) Incidence and Duration of Vasopressor Drugs Used. (8) Incidence of Anti-Antagonist Drugs Used. (9) NRS Score of 24th hour. (10) Incidence of Rescue Analgesics Used. (11) Infusion Volume. (12) Nausea and Vomiting Life Scale Score. (13) Incidence of Abdominal Discomfort. (14) Low Blood Pressure readings from T1 to T7.

### 2.6. Definition of hypotension

Hypotension was defined in this study as SBP below 90 mm Hg or a decrease in MAP of more than 30% from T2 to T7 compared to T1.

### 2.7. Statistical analysis

The significance of differences in primary and secondary outcomes between the 2 groups was assessed using independent samples *t*-tests. A two-sided *P*-value threshold of <.05 was employed to determine statistical significance. Statistical analyses were performed with SPSS 26.0 (IBM, Chicago, IL). Where appropriate, the results were reported together with the corresponding 95% CIs. A *P*-value <.05 in secondary outcomes was interpreted as suggestive. Multiple line graphs were created to visualize the trend of blood pressure values and HR at the 7-timestamps.

## 3. Results

**Figure 1.**
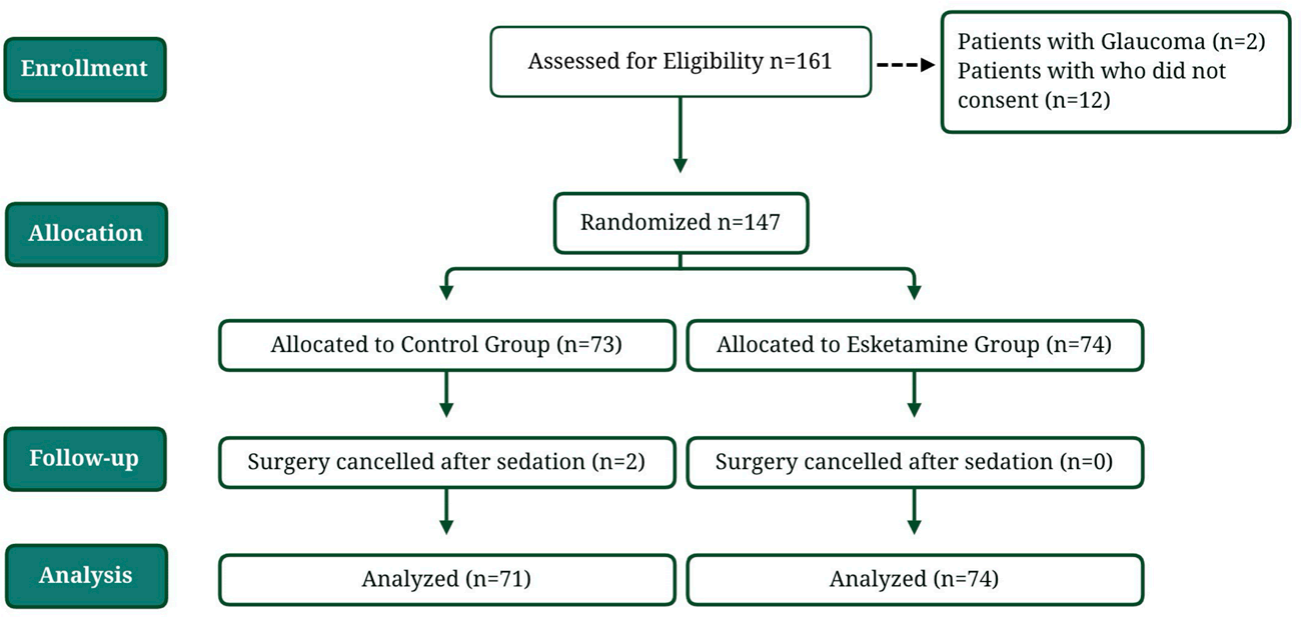
Consort flow diagram.

One hundred sixty-one patients were assessed for eligibility, and 14 of those were excluded. 2 patients had glaucoma, which is an exclusion criterion, and 12 patients did not consent to participate in the study. One hundred forty-seven patients, including 102 females, and 43 males, who were scheduled for bariatric surgery under general anesthesia. The patients were allocated to the control group, n = 73, and the esketamine group, n = 74. 2 patients from the control group had their surgeries canceled after finding gastric contents with endoscopy, leaving 71 patients in the control group and 74 patients in the esketamine group. Data of these 145 patients was then analyzed. A CONSORT flow diagram of trial participants is shown here (Fig. 1). Patients in both groups had similar physical characteristics (Table 1).

**Table 1:** Baseline patient characteristics for all patients and group allocations, data are summarized by number or mean, and the bracket shows standard deviation. Gender, ASA classification, and surgery type data are represented by numbers and the bracket shows percentages.

Characteristic	Control group	Esketamine group	*P*-value
	(n = 71)	(n = 74)	
Age, yr	31.3 (7.9)	33.5 (7.2)	.08
*Sex*			.73
Female	49 (69)	53 (71.6)	
Male	22 (31)	21 (28.4)	
Height, cm	164.2 (7.7)	164.4 (9.4)	.89
Weight, kg	99.5 (17)	98.3 (23.8)	.72
Body mass index, kg·m^−2^	36.9 (5.5)	36 (6.7)	.42
*ASA classification*			.73
II	60 (84.5)	64 (86.5)	
III	11 (15.5)	10 (13.5)	
*Surgery type*			.36
I	64 (90.1)	63 (85.1)	
II	7 (9.9)	11 (14.9)	

ASA = American Society of Anesthesiologists.

### 3.1. Intra and postoperative outcomes

Data collected during and after the operation, surgery duration, extubation time, crystalloid infusion, colloid infusion, vasopressor drugs, vasopressor drugs use duration, anti-antagonist drugs, infusion volume, nausea and vomiting life scale score, and abdominal discomfort showed no statistically significant results *P* > .05 except for 24th hour NRS Score, which was lower in the esketamine group by 0.5 *P* < .05, and the Incidence of Rescue Analgesics Used was lower in the esketamine group by 21.3% *P* < .05. See Table 2.

**Table 2:** Intraoperative data, postoperative data, and group allocations, data are summarized by mean, and the bracket shows standard deviation. Vasopressor drugs, anti-antagonist drugs, and rescue analgesics, and abdominal discomfort data are represented by numbers and bracket shows percentage.

Group	Control group	Esketamine group	*P*-value
Surgery duration, min	87 (20.8)	92.7 (22.4)	.11
Extubation time, min	16.6 (4.2)	17.5 (3.9)	.16
Crystalloid infusion, mL	663.4 (198)	637.2 (202.1)	.43
Colloid infusion, mL	369 (191.7)	339.2 (234.6)	.51
Vasopressor drugs, n	8 (11.3%)	10 (13.5%)	.67
Vasopressor drug use duration, min	7.4 (22.5)	9.7 (27.5)	.59
Anti-antagonist drugs, n	7 (9.9%)	3 (4.1%)	.09
NRS Score (24 h)	2.8 (1.2)	2.3 (1.3)	.03*
Rescue analgesics, n	18 (25.4%)	3 (4.1%)	.00*
Infusion volume, mL	68 (35.8)	74.3 (37)	.30
Nausea and vomiting life scale Score	1.5 (0.8)	1.5 (0.9)	.89
Abdominal discomfort, n	15 (21.1%)	12 (16.2%)	.45

NRS = numerical rating scale.

* *P* < 0.05.

**Figure 2.**
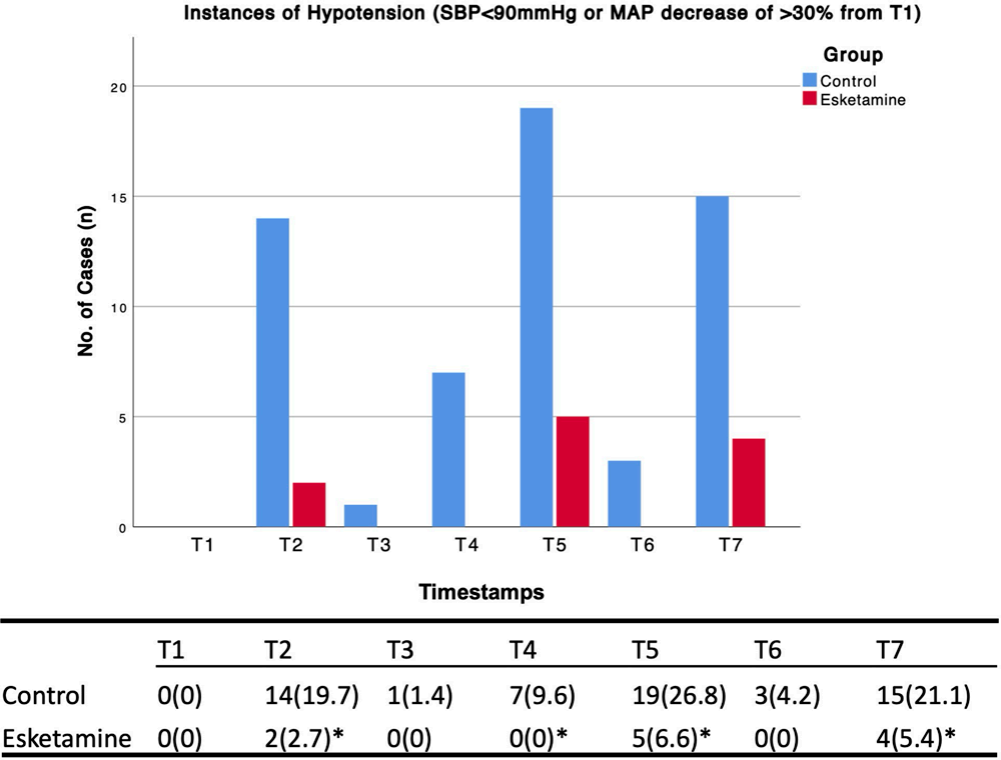
Incidence of low blood pressure from T1 to T7. Timestamps: T1: after dexmedetomidine bolus, T2: right before intubation, T3: 1 minute after intubation, T4: 5 minutes after intubation, T5: Time of incision, T6: 1 minute after incision, T7: 5 minutes after incision. The bracket value is a percentage. * shows significance.

Incidence of hypotension was significantly lower in the esketamine group *P* < .05 at T2 (2.7%), T4 (0%), T5 (6.6%), and T7 (5.4%) compared to control it was 17% lower at T2, 9.6% lower at T4, 20.2% lower at T5, and 15.7% lower at T7. See Figure 2.

**Figure 3.**
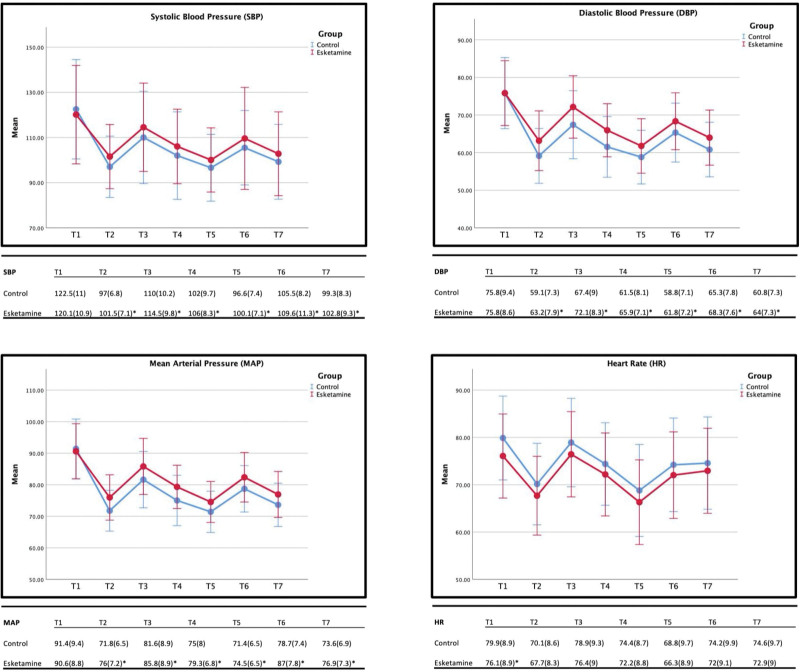
Trend of SBP (systolic blood pressure), DBP (diastolic blood pressure), MAP (mean arterial pressure), and HR (heart rate). Intraoperative data and group allocations, data are summarized by mean, and the bracket shows standard deviation. * shows significance.

SBP, DBP, and MAP were significantly higher in the esketamine group *P* < .05 from T2 to T7 compared to the control group, with an average of 4 mm Hg for SBP, 3.7 mm Hg for DBP, and 4.6 mm Hg for MAP with the largest difference at T6 (8.3 mm Hg). HR in the esketamine group was slightly lower compared to the control group, with a statistically significant decrease of 3.8 bpm *P* < .05 at T1; however, the HR from T2 to T7 showed an insignificant decrease in the esketamine group with an average decrease of 2.6 bpm. See Figure 3.

## 4. Discussion

This study examined the impact of a single 0.2 mg/kg dose of esketamine administered at the beginning of induction on various hemodynamic parameters during bariatric surgery. Our primary outcome measure, blood pressure (SBP, DBP, and MAP) before intubation (T2), showed a statistically significant increase in the esketamine group compared to the control group, consistent with previous research findings.^[23,28]^

Furthermore, we observed that overall blood pressure levels (SBP, DBP, and MAP) from T2 until 5 minutes after incision (T7) were statistically higher in the esketamine group than in the control group. However, it is important to note that the clinical significance of this increase may be limited, potentially due to the relatively low dose of esketamine used in this trial.

Our initial hypothesis was that esketamine administration would lead to a reduction in the incidence of hypotension during the induction of anesthesia. Our findings support this hypothesis, as we observed a significant decrease in the incidence of hypotension at key points, including just before intubation (T2), 5 minutes after intubation (T4), at the time of incision (T5), and 5 minutes after incision (T7). Therefore, we recommend the use of a single dose of 0.2 mg/kg esketamine during the induction phase of bariatric surgery procedures.

Additionally, we found that the NRS scores at the 24th hour and the use of rescue analgesics were also significantly lower in the esketamine group compared to the control group. This finding is consistent with previous studies, some of which employed higher doses of esketamine (0.25 mg/kg, 0.4 mg/kg).^[25,29–31]^

Although HR differences were statistically significant after the administration of dexmedetomidine 0.5 µg/kg for 10 minutes in the esketamine group, these differences were clinically negligible and occurred before esketamine administration. However, HR remained consistently lower in the esketamine group from T2 to T7, with no significant clinical or statistical implications. Other secondary outcomes did not demonstrate clinical or statistical significance.

In summary, our findings suggest that a single dose of 0.2 mg/kg esketamine during the induction phase of anesthesia in bariatric surgery patients can significantly reduce the incidence of hypotension during induction and lead to a slight overall increase in blood pressure. Additionally, it appears to decrease postoperative pain at the 24th hour and reduce the need for rescue analgesics. We recommend routinely administering a single dose of 0.2 mg/kg esketamine during the induction phase of anesthesia in bariatric surgery patients to stabilize blood pressure and prevent the incidence of hypotension during surgery. Future studies may explore the use of higher esketamine doses, considering that this study found that the overall increase in blood pressure was not significant. Importantly, our study implies that administering a low dose of esketamine to hypertensive patients should not pose significant risks to their blood pressure stability, offering potential benefits in terms of pain management and opioid reduction.

Limitations of this study include its single-center nature, which may impact the generalizability of the results, and the predominance of sleeve gastrectomy patients due to its popularity compared to Roux-en-Y bypass surgery.^[32]^ Additionally, BMI criteria for surgery may vary across countries.

## 5. Conclusion

A single dose of 0.2 mg/kg of esketamine does not significantly increase blood pressure; however, it can decrease the incidences of hypotension during the induction phase of anesthesia in bariatric surgery patients, and it may also decrease postoperative pain and reduce the need for rescue analgesics. Future studies should explore higher doses of esketamine.

## Author contributions

**Conceptualization:** Ting Yang.

**Data curation:** Ting Yang, Muhammad Saqib Mudabbar, Mingxing Xu, Qingmei Xiang.

**Formal analysis:** Ting Yang, Muhammad Saqib Mudabbar.

**Funding acquisition:** Qiang Fu.

**Investigation:** Ting Yang, Muhammad Saqib Mudabbar.

**Methodology:** Ting Yang, Muhammad Saqib Mudabbar.

**Project administration:** Ting Yang.

**Software:** Muhammad Saqib Mudabbar.

**Supervision:** Bin Liu, Qiang Fu.

**Writing – original draft:** Ting Yang, Muhammad Saqib Mudabbar.

**Writing – review & editing:** Ting Yang, Muhammad Saqib Mudabbar.

## References

[1] Chooi YC Ding C Magkos F. The epidemiology of obesity. Metabolism. 2019;92:6–10. 30253139 10.1016/j.metabol.2018.09.005

[2] O’Brien PE Hindle A Brennan L. Long-term outcomes after bariatric surgery: a systematic review and meta-analysis of weight loss at 10 or more years for all bariatric procedures and a single-centre review of 20-year outcomes after adjustable gastric banding. Obes Surg. 2019;29:3–14. 30293134 10.1007/s11695-018-3525-0 PMC6320354

[3] Arterburn DE Telem DA Kushner RF. Benefits and risks of bariatric surgery in adults: a review. JAMA. 2020;324:879–87. 32870301 10.1001/jama.2020.12567

[4] Kouz K Wegge M Flick M. Continuous intra-arterial versus intermittent oscillometric arterial pressure monitoring and hypotension during induction of anaesthesia: the AWAKE randomised trial. Br J Anaesth. 2022;129:478–86. 36008202 10.1016/j.bja.2022.06.027

[5] Sharma S Hashmi M Bhattacharya P. Hypotension. 2023. In: StatPearls [Internet]. Treasure Island (FL): StatPearls Publishing. Available at: https://www.ncbi.nlm.nih.gov/books/NBK499961/ [access date February 19, 2023].

[6] Diao S Ni J Shi X. Mechanisms of action of general anesthetics. Front Biosci (Landmark Ed). 2014;19:747–57. 24389218 10.2741/4241

[7] Maheshwari K Turan A Mao G. The association of hypotension during non-cardiac surgery, before and after skin incision, with postoperative acute kidney injury: a retrospective cohort analysis. Anaesthesia. 2018;73:1223–8. 30144029 10.1111/anae.14416

[8] Ackland GL Abbott TEF. Hypotension as a marker or mediator of perioperative organ injury: a narrative review. Br J Anaesth. 2022;128:915–30. 35151462 10.1016/j.bja.2022.01.012 PMC9204667

[9] Lankadeva YR May CN Bellomo R. Role of perioperative hypotension in postoperative acute kidney injury: a narrative review. Br J Anaesth. 2022;128:931–48. 35465952 10.1016/j.bja.2022.03.002

[10] Lizano-Díez I Poteet S Burniol-Garcia A. The burden of perioperative hypertension/hypotension: a systematic review. PLoS One. 2022;17:e0263737. 35139104 10.1371/journal.pone.0263737 PMC8827488

[11] An R Pang QY Liu HL. Association of intra-operative hypotension with acute kidney injury, myocardial injury and mortality in non-cardiac surgery: a meta-analysis. Int J Clin Pract. 2019;73:e13394. 31332896 10.1111/ijcp.13394

[12] Katsimardou A Imprialos K Stavropoulos K. Hypertension in metabolic syndrome: novel insights. Curr Hypertens Rev. 2020;16:12–8. 30987573 10.2174/1573402115666190415161813

[13] Moriconi D Nannipieri M Rebelos E. Bariatric surgery to treat hypertension. Hypertens Res. 2023;46:1341–3. 36813987 10.1038/s41440-023-01227-9

[14] Wang J Huang J Yang S. Pharmacokinetics and safety of esketamine in Chinese patients undergoing painless gastroscopy in comparison with ketamine: a randomized, open-label clinical study. Drug Des Devel Ther. 2019;13:4135–44. 10.2147/DDDT.S224553 PMC6902860 31827320

[15] Fu D Wang D Li W. Pretreatment with low-dose esketamine for reduction of propofol injection pain: a randomized controlled trial. Pain Res Manag. 2022;2022:4289905. 35958679 10.1155/2022/4289905 PMC9363235

[16] Long YQ Feng CD Ding YY. Esketamine as an adjuvant to ciprofol or propofol sedation for same-day bidirectional endoscopy: protocol for a randomized, double-blind, controlled trial with factorial design. Front Pharmacol. 2022;13:821691. 35370640 10.3389/fphar.2022.821691 PMC8975265

[17] Veith SB Nickl R Rössel T. Hemodynamics and cutaneous microcirculation during induction of general anesthesia with and without esketamine. Clin Hemorheol Microcirc. 2023;84:385–98. 37334583 10.3233/CH-231711

[18] Xu Y Zheng Y Tang T. The effectiveness of esketamine and propofol versus dezocine and propofol sedation during gastroscopy: a randomized controlled study. J Clin Pharm Ther. 2022;47:1402–8. 35488787 10.1111/jcpt.13678

[19] Chen Y Chen J Wang Q. Safety and tolerability of esketamine in propofol based sedation for endoscopic variceal ligation with or without injection sclerotherapy: randomized controlled trial. Dig Endosc. 2023;35:845–4. 36808150 10.1111/den.14539

[20] Huang X Ai P Wei C. Comparison of the effects of esketamine/propofol and sufentanil/propofol on the incidence of intraoperative hypoxemia during bronchoscopy: protocol for a randomized, prospective, parallel-group trial. J Clin Med. 2022;11:4587. 35956202 10.3390/jcm11154587 PMC9369459

[21] Liu X Xiao Q Zhuang S. Comparison of propofol–esketamine versus propofol for anesthesia in gastroscopy: a double-blind, randomized controlled clinical trial. Front Med (Lausanne). 2023;10:1184709. 37614948 10.3389/fmed.2023.1184709 PMC10442552

[22] Zhang YY Zhu S Yang X. Esketamine versus sufentanil applied prior to placement of suspension laryngoscope. Laryngoscope. 2023;133:3021–7. 37073819 10.1002/lary.30699

[23] Zhou N Liang X Gong J. S-ketamine used during anesthesia induction increases the perfusion index and mean arterial pressure after induction: a randomized, double-blind, placebo-controlled trial. Eur J Pharm Sci. 2022;179:106312. 36280027 10.1016/j.ejps.2022.106312

[24] Trimmel H Helbok R Staudinger T. S(+)-ketamine. Wien Klin Wochenschr. 2018;130:356–66. 29322377 10.1007/s00508-017-1299-3 PMC6061669

[25] Yang T Mudabbar MS Liu B. Intraoperative esketamine is effective at reducing acute postoperative pain in bariatric surgery patients: a randomized control trial. Obes Surg. 2023;33:2368–74. 37344640 10.1007/s11695-023-06676-2

[26] Kane SP. Sample size calculator: ClinCalc LLC; [cited 2023]. Available at: https://clincalc.com/Stats/SampleSize.aspx.

[27] Doyle DJ Hendrix JM Garmon EH. StatPearls. Treasure Island (FL): StatPearls Publishing Copyright © 2023, StatPearls Publishing LLC.; 2023.

[28] Li J Wang Z Wang A. Clinical effects of low-dose esketamine for anaesthesia induction in the elderly: a randomized controlled trial. J Clin Pharm Ther. 2022;47:759–66. 35018643 10.1111/jcpt.13604

[29] Shen J Song C Lu X. The effect of low-dose esketamine on pain and post-partum depression after cesarean section: a prospective, randomized, double-blind clinical trial. Front Psychiatry. 2022;13:1038379. 36683972 10.3389/fpsyt.2022.1038379 PMC9845877

[30] Ren YL Yuan JJ Xing F. Effects of different doses of esketamine on pain sensitivity of patients undergoing thyroidectomy: a randomized controlled trial. Pain Ther. 2023;12:739–50. 36933139 10.1007/s40122-023-00488-z PMC10199971

[31] Zhang J Wang F Dang J. Effect of intraoperative infusion of esketamine on quality of postoperative recovery in patients undergoing laparoscopic bariatric surgery: a randomized controlled trial. Pain Ther. 2023;12:979–92. 37171754 10.1007/s40122-023-00519-9 PMC10289955

[32] Wu KC Yu EW Schafer AL. Chapter 51 – Skeletal health after bariatric surgery. 2021 2021/01/01/. In: Marcus and Feldman’s Osteoporosis (Fifth Edition). Academic Press; [1261-80]. Available at: https://www.sciencedirect.com/science/article/pii/B9780128130735000514.

